# First Scoring-Event Acquisition Is Associated with Victory in Female Brazilian Jiu-Jitsu: A Bout-Level Analysis of 211 Official Combats

**DOI:** 10.3390/sports14100445

**Published:** 2026-10-09

**Authors:** Marco Antônio Ferreira dos Santos, Naiara Ribeiro Almeida, José Raimundo Fernandes, Michele Andrade de Brito, Diego Valenzuela Pérez, Dany Alexis Sobarzo Soto, Esteban Aedo-Muñoz, Lindsei Brabec Mota Barreto, Ciro Jose Brito

**Affiliations:** 1 Graduate Program of Physical Education, Department of Physical Education, Federal University of Juiz de Fora, Governador Valadares 35010-180, Brazil; marco.dossantos@ufjf.br (M.A.F.d.S.); naiara.ribeiro@ufjf.br (N.R.A.); 2 Vale do Rio Doce University, Governador Valadares 35010-180, Brazil; mundegv@hotmail.com (J.R.F.); michele.psi1980@gmail.com (M.A.d.B.); 3 Facultad de Salud y Ciencias Sociales, Escuela de Kinesiología, Universidad de las Américas, Santiago 7500912, Chile; dvalenzuelap@udla.cl; 4 Facultad de Salud, Escuela de Kinesiología, Universidad Santo Tomás, Puerto Montt 5480000, Chile; danysobarzo@santotomas.cl; 5 Deportes y Recreación, Escuela de Educación Física, Universidad Metropolitana de Ciencias de la Educación, Santiago 7760197, Chile; esteban.aedo@umce.cl; 6 Graduate Program in Food, Nutrition, and Health (PPGANS), Research Center for Sports Performance, Combat Sports and/or Martial Arts (DECLAM), Faculty of Education, Federal University of Grande Dourados, Dourados 79825-070, Brazil; lindseibarreto@ufgd.edu.br; 7 Graduate Program of Physical Education, Federal University of Viçosa, Campus Florestal, Florestal 35690-000, Brazil

**Keywords:** female athletes, technical-tactical analysis, combat sports, competitive success, early advantage

## Abstract

Objective: To examine the association between first scoring-event acquisition and subsequent victory in female Brazilian jiu-jitsu (BJJ), accounting for paired opponents and repeated tournament appearances. Methods: This secondary analysis included 211 bouts from the 2020 IBJJF Pan American Championship, representing 286 unique athletes. First scoring was defined as the first positive point-scoring action recorded during the bout; scoreless bouts, including those decided solely by advantages, were excluded from the primary analysis. Conditional logistic regression stratified by bout estimated the primary association, with a paired-comparison mixed-effects model used as a sensitivity analysis for repeated athlete appearances. Technique conversion was compared by chi-square, and timing by Kruskal–-Wallis with Dunn–-Holm post hoc tests. Results: An identifiable first- scoring event occurred in 180/211 bouts (85.3%). The first-scoring athlete won 157/180 bouts (87.2%; 95% CI 81.6–91.3%). First scoring was associated with higher odds of victory (conditional OR = 6.83, 95% CI 4.41–10.57; *p* < 0.001). Guard passes resulted in 57/61 wins (93.4%), sweeps in 57/66 (86.4%), and takedowns in 35/45 (77.8%). Technique conversion did not reach statistical significance (χ^2^ = 5.49, df = 2, *p* = 0.064; Cramer’s V = 0.179). Timing differed among techniques (H = 40.23, *p* < 0.001), with takedowns occurring earliest (median 15 s; IQR 10–41). Conclusions: First scoring was strongly associated with subsequent victory in female BJJ, although causality cannot be inferred from this observational design. Technique-specific conversion showed an exploratory pattern, and takedowns occurred earlier than ground-based scoring actions.

## 1. Introduction

Brazilian jiu-jitsu (BJJ) is a technically and tactically complex grappling sport in which competitive performance depends on positional control, continuous transitions, submission opportunities, and the accumulation of points or advantages over the course of the bout [1,2]. This complexity is reflected in the wide range of motor interactions and tactical pathways available to athletes, including guard control and breaking, passing, inversions, dominant positions, escapes, and submissions [3,4,5]. Competitive success therefore depends not only on the execution of isolated techniques, but also on how athletes sequence these actions to convert standing exchanges into advantageous ground situations, sustain positional dominance, and create effective scoring or finishing opportunities [3,6]. This sequential nature is also reflected in the official scoring system. Under the IBJJF rules, takedowns and sweeps score two points, guard passes score three, and mount or back-control positions score four (International Brazilian Jiu-Jitsu Federation) [3,5,7]. The scoring structure formalizes a competitive logic in which athletes may pursue submission while also managing cumulative points and advantages [8]. Because scoring actions emerge through positional development rather than isolated exchanges, the timing and order of these events may be relevant to competitive outcome. In this context, the first scoring event establishes an initial score state that may subsequently be defended, reversed, or extended [9,10].

The potential importance of early scoring is supported by evidence from other combat sports, where obtaining an early scoring or positional advantage has been associated with a higher probability of victory, although the strength of this association varies according to the sport and ruleset [11,12]. In judo, early penalties can place athletes at a disadvantage, disrupt initiative, and alter the path to decisive scoring, while groundwork scoring often emerges from prior tactical sequences rather than isolated actions [13]. In other combat sports, success also reflects early or pre-existing competitive advantages, including stronger finishing profiles and bout-day weight differentials in MMA and boxing [14,15]. However, this evidence derives predominantly from male samples, and BJJ differs from these sports because its continuous positional progression and cumulative scoring system allow early advantages to be defended, reversed, or amplified over time rather than functioning as a simple first-strike effect [6]. Accordingly, whether the first point carries a meaningful and consistent competitive advantage in BJJ remains an empirical question that should be examined directly using systematically coded official match data rather than inferred from evidence in other combat sports [8,13].

This question is particularly relevant in female BJJ, where athletes remain under-represented in combat-sport research. Female-specific studies from this research program have examined time–motion structure, weight divisions, winner–loser profiles, belt graduation, and adult-versus-master technical–tactical behavior using the same underlying 2020 Pan-American competition cohort [3,9,16]. Two recent publications specifically examined belt graduation [17] and adult-versus-master technical–tactical differences [18]. Together with the earlier reports from the same cohort, these studies make the provenance of the dataset explicit and distinguish what has already been published from the present contribution. None of those publications used a bout-matched framework to examine first scoring-event acquisition, lead conversion, and subsequent outcome while also accounting for repeated tournament appearances. The present study is therefore a secondary analysis of an established dataset addressing a distinct scoring-sequence question. Because first scoring may reflect both an in-bout score state and pre-existing between-athlete competitive superiority, the design and interpretation are explicitly associative rather than causal.

Addressing this gap requires an observational approach capable of capturing not only the occurrence of scoring actions but also their technical classification and temporal sequence. FRAMI-based protocols have been developed and validated to classify combat phases, technical actions, scoring-related events, and temporal features systematically [17]. Therefore, this study aimed to examine whether acquisition of the first scoring event was associated with subsequent victory in official female BJJ competition using a bout-level matched framework. Secondary objectives were to describe technique-specific conversion and compare timing across technique categories. We hypothesized that (i) the first-scoring athlete would show a higher probability of subsequent victory; (ii) victory conversion would differ across the three principal first-scoring techniques; and (iii) the timing of the first scoring event would differ across technique categories, with takedowns occurring earlier than ground-based scoring actions.

## 2. Materials and Methods

### 2.1. Study Design and Ethical Considerations

This retrospective cross-sectional observational study was a secondary analysis of a previously established 2020 IBJJF Pan American Championship dataset. The study followed the Declaration of Helsinki and the Strengthening the Reporting of Observational Studies in Epidemiology (STROBE) guidelines [19]. The original project used secondary data from publicly accessible competition footage and records, without intervention or participant contact. In accordance with Article 1, sole paragraph, item III, of Brazilian National Health Council Resolution No. 510 of 7 April 2016, the original study was considered outside mandatory CEP (Research Ethics Committee) and CONEP (National Research Ethics Commission) review for research using public-domain information. Athlete names available in public competition materials were retained only in the protected working file for record linkage and identification of repeated tournament appearances; names are not reported in publications or shared datasets and were replaced by anonymized athlete_id values for the present revision.

### 2.2. Participants, Competition Settings, and Sampling Framework

The analysis used the complete available female cohort from the 2020 IBJJF Pan American Championship, comprising 211 official combats and 422 athlete-performance records (two records per combat). These 422 records must not be interpreted as 422 independent athletes. After identity reconciliation, they represented 286 unique athletes: 171 appeared once, 96 twice, 17 three times, and 2 four times. Thus, 115 athletes contributed more than one combat. The present sample fully overlaps the underlying competition dataset originally; prior publications from this research program addressed different technical–tactical questions using the same cohort. The current hypotheses and inferential analyses concern first scoring-event acquisition, lead conversion, technique, and timing.

Eligible combats were official female IBJJF matches from the Pan American Championship held in Kissimmee, Florida, USA, in October 2020, with complete analyzable high-definition video recordings, clearly identifiable combat outcomes, and complete information on first-point acquisition, scoring technique, and scoring time. In the primary analysis, first scoring was defined exclusively as the first positive point-scoring action recorded during the bout. Point-scoreless bouts, including those decided by a greater advantage total, were not classified as having a first scoring event and were excluded from the primary first-scoring analysis. Bouts decided solely by advantages were examined and reported separately. All belt levels, weight categories, and tournament phases represented in the competition were eligible, resulting in 180 bouts with an identifiable first positive point-scoring event and 31 without one in the final primary analysis. Four first-scoring events were not classified within the three principal technique categories used for the omnibus comparison and were therefore excluded from that subgroup analysis; timing data were available for 178 of the 180 first-scoring events. Combats were excluded if they were interrupted by injury, medical problems, or equipment failure; decided by disqualification; had incomplete, unclear, or disputed scoring information; were decided exclusively by penalties without positive technical scoring; or were exhibition, demonstration, or superfight bouts. Two isolated first-scoring status fields were initially missing. During the data audit, the opponent in each corresponding bout was confirmed as the first scorer, and the missing fields were therefore reconciled as “No.” The audit also identified four additional first-scoring classifications requiring correction: one bout in which a positive 9-point score was identified (bout 144), and three point-scoreless bouts initially classified as first-scoring because a greater advantage total directly determined the outcome (bouts 82, 176, and 207). The three advantage-only bouts were subsequently excluded from the primary first-scoring analysis. All corrections were documented in the change log. Thus, the final dataset contained 422 athlete-performance records representing 211 bouts, with first-scoring status available for all records. Bouts without an identifiable first positive point-scoring event (n = 31) were described separately and did not contribute to the first-scorer conversion analysis.

### 2.3. Data Collection and Outcome Definitions

Combat data were extracted from official high-definition IBJJF competition recordings using the FRAMI software 2.0 (Framework for Analysis of Mixed Martial Arts and Grappling Sports) and its validated BJJ combat analysis protocol [20]. First-scoring status, the technique producing the first score, and the timing of that event were not introduced retrospectively for the present study. These variables were already recorded and analyzed in the original competition dataset as part of the FRAMI-based technical–tactical assessment, in which scoring actions and their temporal occurrence were systematically coded.

The FRAMI protocol had previously undergone intra- and inter-evaluator reliability assessment; however, no new reliability coefficient restricted specifically to these three variables was calculated for the complete 211-bout dataset.

The protocol recorded first-scoring status, the technical category of the first scoring action, scoring time, and combat outcome. The original validation involved four evaluators: three experts and one non-expert. The expert master was a black belt with approximately 10 years of practice, more than five years of teaching experience, international competitive experience, and international referee qualification; the other experts included a black belt and a brown belt with more than seven years of practice and national competitive/teaching experience; the non-expert was a purple belt with less than five years of practice. Evaluators completed a seven-day familiarization period, and the protocol was examined using test–retest, intra- and inter-evaluator procedures, intraclass correlation, Cronbach alpha, and Bland–Altman analyses. The validation reported high overall reliability (alpha = 0.90; agreement coefficients approximately 0.78–0.97).

Because first scoring and its timing were prespecified components of the validated protocol, the present study treats them as protocol-defined observations rather than newly derived post hoc variables. Although a separate reliability coefficient specific to these variables was not calculated for the present 211-bout sample, this limitation is stated explicitly. For the present analysis, first-point acquisition was defined as the first official positive point-scoring action recorded during the combat; advantage-only decisions were not considered first-point events. Scoring time was measured from the official start signal to the referee’s scoring signal and recorded to the nearest 0.1 s. First-point techniques were classified as takedowns, guard passes, sweeps, or dominant-position acquisitions. Takedowns included projection techniques resulting in ground control; guard passes included actions that progressed beyond the opponent’s guard to a dominant position; sweeps included reversals initiated from guard positions that resulted in top control; and dominant-position acquisitions included direct attainment of the mount, back control, technical mount, or knee-on-belly. Victory was classified as submission, points, advantage, or referee decision.

### 2.4. Statistical Analysis

Statistical analyses were reconstructed from the anonymized audited dataset using Python 3.9.7 with pandas 1.5.2, NumPy 1.24.1, SciPy 1.10.0, scikit-learn 1.2.0, and statsmodels 0.13.5. The combat was the primary inferential unit. For bouts with an identifiable first positive point-scoring event, the primary descriptive estimate was the proportion in which the first-scoring athlete subsequently won, with a Wilson 95% confidence interval. The primary association was estimated using conditional logistic regression stratified by bout, thereby comparing the two opponents within the same combat and respecting their structural dependence. Only bouts with one athlete scoring first and the opponent not scoring first contributed to estimation of the first-scoring coefficient.

Because some athletes appeared in multiple tournament bouts, a paired-comparison mixed-effects model analogous to a Bradley–Terry framework was fitted as a sensitivity analysis. Athlete identity was represented by the anonymized athlete_id variable, and athlete-specific random intercepts were included to account for repeated athlete appearances across bouts. The model retained the paired structure of each combat, with the two athlete-performance records representing the opposing competitors, and included first-scoring status as the fixed-effect predictor of victory. The coefficient for first-scoring status was exponentiated to obtain the odds ratio (OR). Uncertainty for the sensitivity estimate was summarized as an approximate 95% credible interval on the odds-ratio scale, with the interval limits obtained by exponentiating the corresponding limits on the model coefficient scale.

Technique-specific victory conversion was evaluated among classified first-scoring techniques. The prespecified comparison among takedowns, sweeps, and guard passes used a Pearson chi-square test with Cramer’s V reported as an omnibus effect-size measure; sparse other scoring events were reported descriptively. Because first-event times were positively skewed with substantial outliers, timing was summarized as median [IQR] and compared using Kruskal–Wallis tests followed by Dunn pairwise comparisons with Holm multiplicity adjustment. As an additional sensitivity analysis addressing the mechanical contribution of points to final score, first-scorer conversion was stratified by method of victory; points versus submission strata were compared with Fisher’s exact test. Alpha = 0.05 was prespecified as the decision threshold for formal hypothesis testing. However, *p* values were interpreted together with effect estimates, confidence intervals, and sample size rather than as a binary measure of scientific importance; values just above 0.05 were not treated as evidence of equivalence or absence of an effect, consistent with contemporary statistical guidance [21].

## 3. Results

### 3.1. Sample Characteristics

The final sample comprised 211 official female bouts from the 2020 IBJJF Pan American Championship, corresponding to 422 athlete-performance records. After reconciliation of spelling and truncation variants used solely for record linkage, 286 unique athletes were identified. Of these, 171 athletes contributed one bout, 96 contributed two bouts, 17 contributed three bouts, and 2 contributed four bouts. Each reconciled bout contained exactly two opponent records and one winner. The distinction between bouts, athlete-performance records, and unique athletes was maintained throughout all analyses. Blue belt athletes represented the largest proportion of the sample (35.5%), followed by purple (25.6%), white (14.2%), brown (13.3%), and black (11.4%) belts. Adults aged 18–29 years accounted for 59.2% of the sample, while masters aged ≥30 years accounted for 40.8%. All eight IBJJF weight categories and all tournament phases were represented.

### 3.2. First-Point Acquisition and Victory

**Figure 1:**
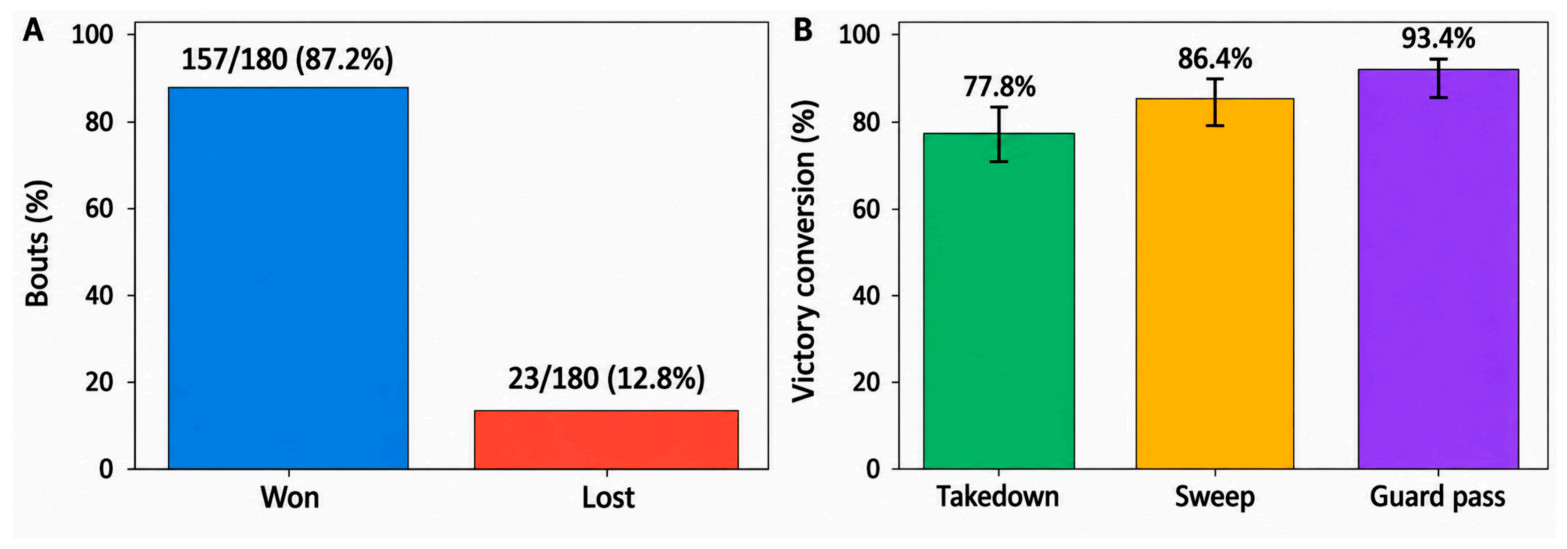
(**A**) Outcomes of the 180 bouts with an identifiable first scoring event. (**B**) Victory-conversion rates for the three principal first-scoring techniques, with 95% confidence intervals. The omnibus comparison yielded χ^2^ = 5.49, df = 2, *p* = 0.064, with Cramer’s V = 0.179.

An identifiable first-scoring event occurred in 180 of 211 bouts (85.3%), whereas 31 bouts (14.7%) had no identifiable positive point-scoring event. Among the 180 bouts with an identifiable first scoring event, the athlete who scored first won 157 bouts and lost 23, corresponding to a victory rate of 87.2% (95% CI 81.6–91.3%). The opponent won 23 of these bouts (12.8%). Among the classified conventional first-scoring techniques, guard passes resulted in 57 wins in 61 bouts (93.4%; 95% CI 84.3–97.4%), sweeps in 57 of 66 bouts (86.4%; 95% CI 76.1–92.7%), and takedowns in 35 of 45 bouts (77.8%; 95% CI 63.7–87.5%). The omnibus comparison yielded χ^2^ = 5.49, df = 2, *p* = 0.064, with Cramer’s V = 0.179 (Figure 1).

### 3.3. Temporal Characteristics of First-Point Acquisition

**Figure 2:**
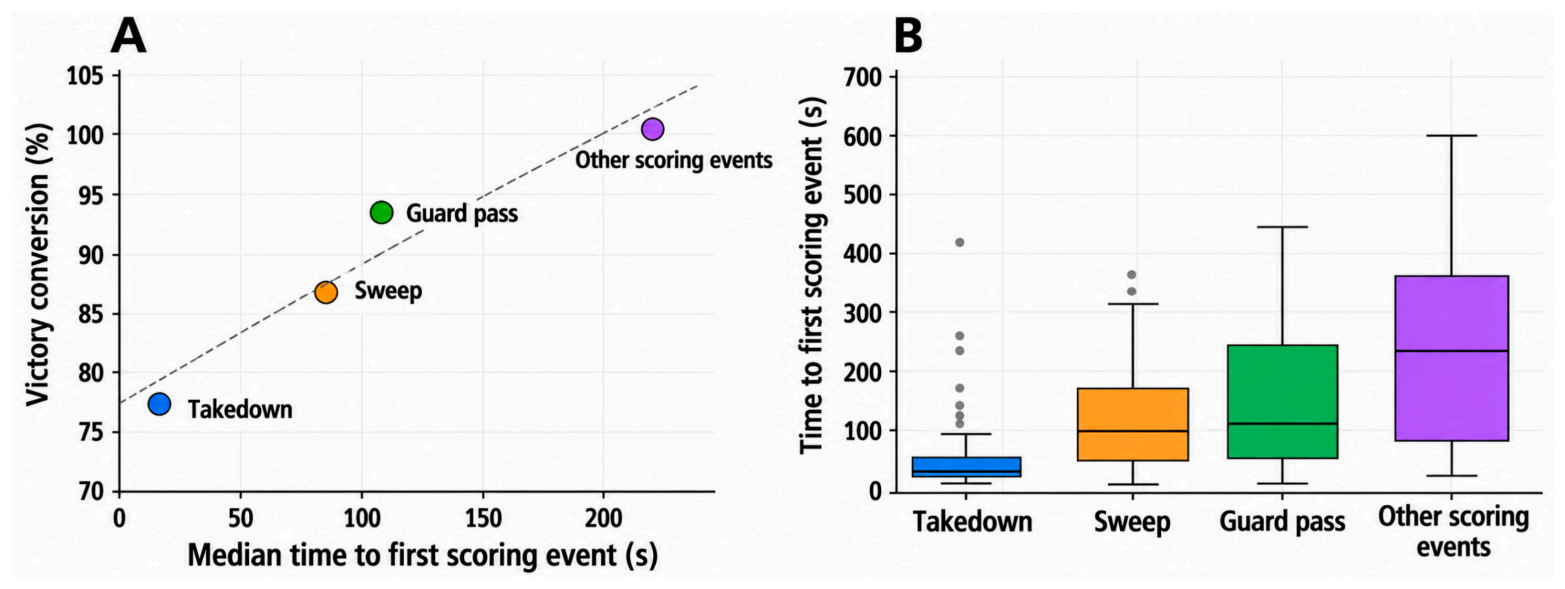
Temporal characteristics of the first scoring event by technique group. (**A**) Relationship between median time to the first scoring event and victory conversion rate across technique groups. (**B**) Distribution of time to the first scoring event by technique group; boxes show the median and interquartile range, whiskers extend according to the standard 1.5 × IQR convention, and grey dots represent individual outlier observations beyond 1.5 × IQR. Inference for timing differences used the Kruskal–Wallis test followed by Dunn–Holm pairwise comparisons.

Timing data were available for 178 classified first-scoring events. Distributions were positively skewed; accordingly, medians [IQR] are reported. Takedowns occurred at 15.0 [10.0–41.0] s (*n* = 45), sweeps at 85.0 [36.8–150.8] s (n = 66), guard passes at 107.5 [47.0–220.0] s (n = 60), and other scoring events at 220.0 [61.0–335.0] s (n = 7). The overall difference was significant (Kruskal–Wallis H = 40.23, df = 3, *p* < 0.001). Dunn–Holm tests showed that takedowns occurred earlier than sweeps (adjusted *p* = 0.000031), guard passes (adjusted *p* < 0.000001), and other scoring events (adjusted *p* = 0.000731). Sweep versus guard pass and the remaining pairwise comparisons were not statistically significant after Holm adjustment (Figure 2).

### 3.4. Matched Association and Repeated-Athlete Sensitivity Analysis

**Figure 3:**
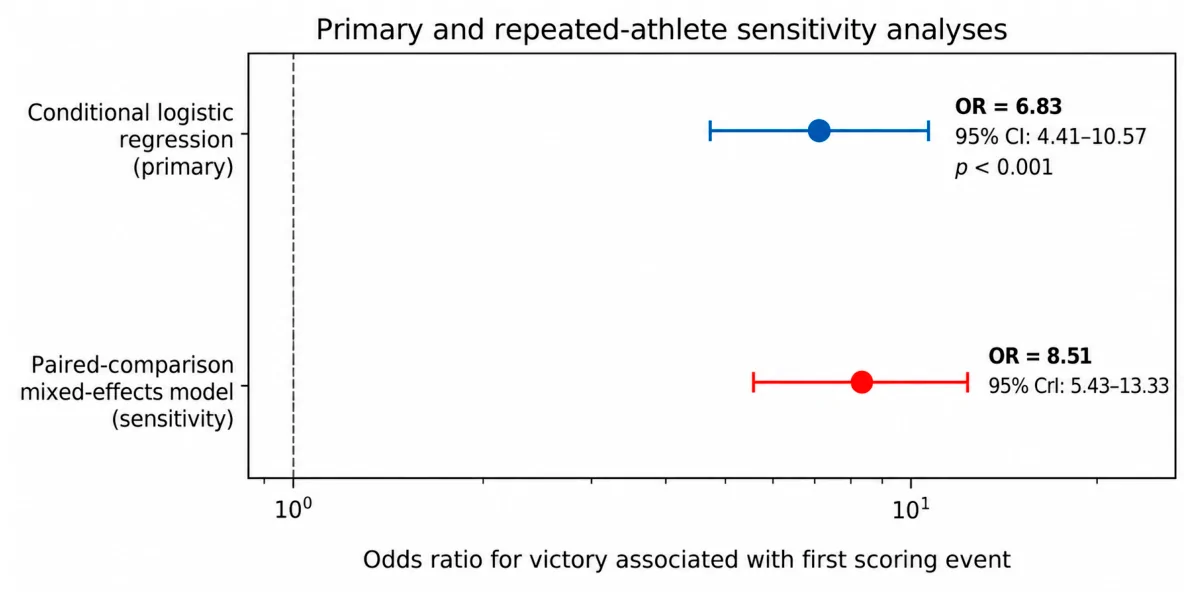
Association between first scoring-event acquisition and subsequent victory in the primary bout-stratified conditional logistic regression and the repeated-athlete paired-comparison mixed-effects sensitivity model. Points represent odds ratios, and horizontal bars represent the corresponding uncertainty intervals. The primary and sensitivity estimates are shown in blue and red, respectively. The sensitivity interval is an approximate 95% credible interval.

Conditional logistic regression stratified by bout showed that acquisition of the first scoring event was associated with higher odds of subsequent victory (OR = 6.83, 95% CI 4.41–10.57; *p* < 0.001). The paired-comparison mixed-effects sensitivity model, incorporating athlete-specific random effects to account for repeated tournament appearances, yielded an OR of 8.51 (approximate 95% credible interval 5.43–13.33). In the method-of-victory sensitivity analysis, first-scorer conversion was 98/109 (89.9%) among bouts won by points and 56/67 (83.6%) among bouts won by submission. The Fisher’s exact test yielded OR = 1.75, *p* = 0.245 (Figure 3).

## 4. Discussion

The present study indicates that the acquisition of the first positive scoring event was strongly associated with subsequent victory, with the same general pattern persisting across the paired and sensitivity analyses. This association should not, however, be interpreted as evidence that scoring first independently causes victory. Rather, it may reflect a combination of the effects of the competitive state following the initial score and underlying differences in competitors’ technical–tactical ability. The latter interpretation is particularly relevant because, under BJJ scoring rules, an early score can directly contribute to the final point differential. In the sensitivity analysis by method of victory, first-scorer conversion was 89.9% among bouts won by points and 83.6% among bouts won by submission, with no statistically significant difference between these strata (Fisher’s exact test, *p* = 0.245). Nevertheless, the persistence of the association among bouts ultimately decided by submission, where no point differential determined the outcome, suggests that the first scoring event may represent more than a simple arithmetic component of the final result. It may therefore serve as a useful marker of competitive advantage, while its causal role remains uncertain in this observational sample. The technique-specific findings should likewise be interpreted cautiously. Although conversion rates varied across takedowns, sweeps, and guard passes, the evidence did not meet the prespecified threshold for a confirmatory between-technique effect. At the same time, the absence of statistical significance should not be interpreted as evidence that the techniques are equivalent. Consistent with current guidance on the interpretation of *p* values, the observed pattern may be considered exploratory and hypothesis-generating rather than definitive [21]. Replication in larger samples will be necessary to determine whether the apparent differences reflect a reproducible technique-specific pattern.

The revised analysis does not infer absence of effect modification merely because age category, belt, weight category, or competition phase failed to show independent main effects in the former ordinary logistic model. Those variables are shared bout characteristics or tournament descriptors and do not substitute for athlete-specific competitive ability. Accordingly, claims that the association was universally robust across all strata have been removed. Future datasets with larger numbers of bouts within each stratum can test explicit interactions and athlete-level moderators prospectively. Previous research has emphasized the importance of technical–tactical interactions and positional behavior in determining BJJ performance [3,8,16].

The association between early scoring and victory is also consistent with findings from other combat sports, where competitive success has been related to technical–tactical and temporal characteristics of combat, although the underlying mechanisms and scoring systems differ across modalities [22,23,24]. In BJJ, continuous ground engagement and cumulative scoring may contribute to the maintenance of an initial advantage through positional control rather than requiring an immediate decisive action [25]. The relevance of the first point may therefore be better understood as part of an evolving sequence of positional interactions rather than as an isolated action. Time–motion studies showing different technical and behavioral patterns between winning and losing BJJ athletes further support the broader role of positional and tactical regulation during combat [3,16,25]. The technique-specific findings should likewise be interpreted cautiously. Although victory-conversion rates varied numerically across takedowns, sweeps, and guard passes, the evidence did not meet the prespecified threshold for a confirmatory between-technique effect. A successful sweep simultaneously reverses the positional hierarchy and places the athlete in top control, creating immediate access to pressure-based scoring sequences, which may contribute to its high conversion despite occurring later in the combat [6,8,26]. Guard passing, in turn, removes an important defensive structure while scoring, opening access to dominant top positions and subsequent scoring or submission opportunities [6,26]. Takedowns, in contrast, occur before ground exchanges have developed and appear to function primarily as an entry into an advantageous starting position from which subsequent control must still be constructed [8,9,27]. The temporal ordering observed among techniques is consistent with these distinct tactical prerequisites, as takedowns tend to occur earlier, whereas guard-passing and other ground actions occur later in the contest [8,26]. Finally, first points obtained through direct acquisition of dominant positions were rare and were associated with victory in all observed cases; however, the small number of such events warrants caution, particularly because these actions appear less frequently than guard passing and takedown actions in available notational studies [27,28].

Experience may broaden an athlete’s technical repertoire and improve tactical adaptability [29]. Previous research has demonstrated differences in technical and tactical behavior according to experience and competitive level in BJJ and related combat settings, including differences in guard-pass variety, competitive approach, and defensive behavior [8,11,20]. At the same time, some combat-determining phases, such as gripping, transition, side control, and mount, have not differed clearly across female belt levels, suggesting that differences in competitive experience do not necessarily alter the structural importance of decisive positional actions [20]. Weight-category differences in BJJ have likewise been more consistently expressed through activity profiles, gripping duration, attack time, and effort distribution than through clear differences in outcome-defining tactical structures [3,30].

The high conversion rate observed among athletes who acquired the first scoring event indicates a strong association between the initial scoring state and subsequent victory. Nevertheless, this association should not be interpreted as deterministic or as evidence of a causal effect, as a proportion of athletes who conceded the first point still won their combats. Competitive BJJ performance is inherently multifactorial, involving technical–tactical behavior, physical capacities, opponent interactions, and multiple possible action sequences [28,31]. Accordingly, first-point acquisition should be interpreted as one component of a broader competitive process rather than as a standalone determinant of victory. Its relevance may depend on the context in which the score is obtained and, particularly, on the actions that follow it [9,26].

From a practical perspective, the findings support incorporating first-scoring scenarios into technical–tactical preparation. Training may benefit from integrating opening-phase takedown sequences, guard-passing strategies, and structured responses to conceding the first point, while preserving positional control and avoiding unnecessary risk. For performance analysts, the combination of first-scoring technique, timing, and subsequent scoring sequence may provide additional contextual information beyond first-point status alone. Validated observational systems such as FRAMI can facilitate the systematic identification of these patterns in competitive settings [20]. Importantly, the present findings should not be interpreted as evidence that athletes should pursue the first score at the expense of tactical stability; rather, they suggest that early scoring is a relevant component of the competitive process that warrants specific attention in preparation and analysis.

### 4.1. Limitations

Some limitations should be considered. First, the observational design prevents causal inference and cannot distinguish the association between obtaining the first scoring event and subsequent victory from underlying athlete ability. Second, all data derive from a single event—the 2020 IBJJF Pan American Championship—which provides contextual consistency but limits external validity across seasons, federations, and rulesets. Third, repeated athletes were present; although the sensitivity model incorporated athlete-specific heterogeneity, ranking, prior competitive success, training history, and opponent quality were unavailable. Fourth, 31 bouts had no identifiable first positive point-scoring event under the revised definition and were described separately, including three bouts decided solely by advantages; four additional bouts with an identifiable first scoring event could be classified for the primary outcome analysis but lacked conventional technique/timing information and were excluded from those subanalyses. Fifth, the original protocol was validated with test–retest and inter/intra-evaluator procedures and first-scoring tagging was a prespecified protocol variable, but a new reliability study restricted solely to first-event technique and timing was not conducted on the entire 211-bout dataset. Finally, the study uses one competition dataset previously employed for other technical–tactical questions; the current contribution is therefore a transparent secondary analysis rather than an independent cohort replication.

### 4.2. Practical Applications

From a practical perspective, the findings support monitoring the first scoring event, the subsequent preservation or reversal of the lead, and the athlete’s response after conceding. Coaches may use these patterns to design hypothesis-informed training scenarios involving opening scoring exchanges and score-state management. However, the present study did not test a training intervention, so it cannot demonstrate that training athletes to score first will causally increase the probability of victory.

## 5. Conclusions

The first positive point-scoring event was strongly associated with subsequent victory in BJJ contests, suggesting that first scoring may serve as a marker of competitive advantage. Technique-specific conversion rates showed variation, but the evidence was insufficient to support a definitive difference between techniques. Overall, the findings support the potential relevance of first scoring within the competitive process, while supporting further research to determine whether this association reflects a causal effect or underlying differences in competitors’ technical–tactical ability.

## Data Availability

The data supporting the findings of this study are publicly available in Figshare: https://doi.org/10.6084/m9.figshare.33306663.

## Abbreviations

The following abbreviations are used in this manuscript:

BJJ	Brazilian Jiu-Jitsu
CAPES	Coordenação de Aperfeiçoamento de Pessoal de Nível Superior
CI	Confidence Interval
df	Degrees of Freedom
FRAMI	Framework for Analysis of Mixed Martial Arts and Grappling Sports
IBJJF	International Brazilian Jiu-Jitsu Federation
IQR	Interquartile Range
MMA	Mixed Martial Arts
OR	Odds Ratio
STROBE	Strengthening the Reporting of Observational Studies in Epidemiology
